# Comparative efficacy between retrograde intrarenal surgery with vacuum-assisted ureteral access sheath and minimally invasive percutaneous nephrolithotomy for 1–2 cm infectious upper ureteral stones: a prospective, randomized controlled study

**DOI:** 10.3389/fsurg.2023.1200717

**Published:** 2023-07-07

**Authors:** Qing-lai Tang, Ping Liang, Ye-fei Ding, Xing-zhu Zhou, Rong-zhen Tao

**Affiliations:** ^1^ Department of Urology, The Affiliated Jiangning Hospital with Nanjing Medical University, Nanjing, China; ^2^ Department of Hepatobiliary Surgery, The Second Hospital of Nanjing, Nanjing, China; ^3^ Department of Urology, Liaocheng People’s Hospital, Liaocheng, China

**Keywords:** retrograde intrarenal surgery, minimally invasive percutaneous nephrolithotomy, vacuum-assisted ureteral access sheath, upper ureteral stone, infectious

## Abstract

**Objective:**

To observe the efficacy and safety of retrograde intrarenal surgery combined with vacuum-assisted ureteral access sheath (V-UAS) and minimally invasive percutaneous nephrolithotomy (MPCNL) in patients with 1–2 cm infectious upper ureteral stone.

**Patients and methods:**

A total of 173 patients with 1–2 cm infectious upper ureteral stone were prospectively randomized into two groups. Eighty-six in the V-UAS group and 87 cases as control in the MPCNL group. The SFRs at different times (Postoperative 1 day, 2nd week and 4th week) was considered as the primary outcome of the study. The secondary end points were operative time, postoperative hospital stay and operative complications.

**Results:**

There was no obvious difference between two groups in patients' demographics and preoperative clinical characteristics (all *P *> 0.05). Postoperative data showed that the SFR at postoperative 1 day in the V-UAS group was significantly lower than that in the MPCNL group (73.2% vs. 86.2%, *P *= 0.034). However, there was no statistical significance between two groups in SFRs during postoperative 2 weeks and 4 weeks (All *P* > 0.05). The levels of WBC, CRP and PCT were all significant lower in the V-UAS group than those in the MPCNL group at the postoperative 24 h and 48 h (all *P *< 0.05). Postoperative complications included fever (≥38.5°C), bleeding, pain and urosepsis. In terms of the rates of fever, pain and urosepsis, MPCNL group were all significantly higher than those in the V-UAS group (10.3 vs. 2.4%, *P* = 0.031; 14.9 vs. 2.4%, *P* = 0.003; 4.6 vs. 0.0%, *P* = 0.044; respectively). No significant difference was found between two groups in bleeding. Meanwhile, postoperative hospital stay in the V-UAS group was more shorten than that in the MPCNL group (3.7 vs. 5.9 days, *P *< 0.001).

**Conclusions:**

Our study showed that RIRS with V-UAS, a new partnership to treat 1–2 cm infectious upper ureteral stones, was satisfying as it achieved a high SFR rate and a low rate of infectious complications. This method was safe and reproducible in clinical practice.

## 1. Introduction

Upper ureteral stones with urinary tract infection (UTI) are often encountered in urological practice, more commonly in females (10%–11% vs. 4% in males) and older adult patients (1, 2). Although the incidence has decreased over the last 30 years due to increasingly improving medical care, upper ureteral stones with UTI often lead to episodes of renal colic, renal insufficiency, systemic inflammatory response syndrome (SIRS), or urosepsis if not effectively treated.

Urolithiasis might develop as a result of UTI, whereas non-infectious stones, originating from metabolic disturbances or unknown physiopathologic changes, can cause UTI in reverse (3). Surgery aiming for complete stone removal is the treatment mainstay, and minimally-invasive percutaneous nephrolithotomy (MPCNL) is the treatment of choice for most upper ureteral 1–2 cm stones with UTI according to the American Urological Association guidelines (4). It was reported that the stone-free rate (SFR) could reach 78%–95% after MPCNL (5, 6). However, these patients are always at an elevated risk for potentially serious complications, including fever, bleeding, urosepsis, and even death (7). In the recent decade, retrograde intrarenal surgery (RIRS) was widely utilized to treat upper ureteral or renal stones with the advantages of being less invasive, with less hemorrhage, and a shorter hospital stay. However, intrarenal pressure control and residual fragments remain two major drawbacks of RIRS (8, 9). An intrarenal pressure over 40 mmHg might cause pyelovenous backflow, which is likely to aggravate UTI, especially with infectious upper ureteral stones (10). Conversely, discharge of residual fragments is a self-elimination time-dependent process that might lead to renewed infection or obstruction of the urinary tract (11, 12). In a study of 384 patients undergoing RIRS, abdominal computed tomography performed 3–12 weeks postoperatively detected clinically insignificant residual fragments in 44 patients (11.5%). Among them, 15 showed symptoms resulting from the enlargement or fusion of residual fragments (13). Therefore, it is essential to promote residual fragment discharge from the urinary tract as soon as possible following lithotripsy.

**Figure 1:**
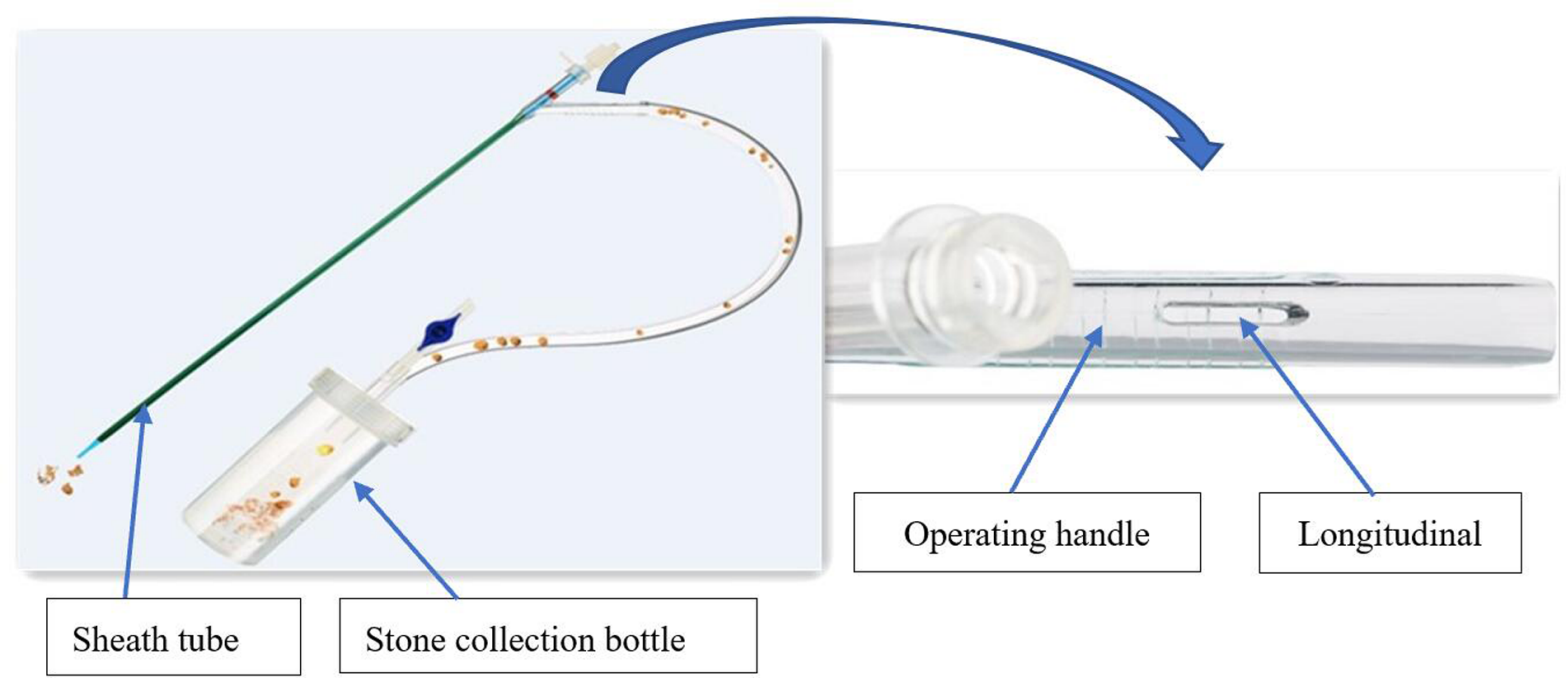
Diagram for vacuum-assisted ureteral access sheath.

Recently, our urological department started using a novel disposable vacuum-assisted ureteral access sheath (V-UAS, Y-type, Wellead Medical, Guangzhou, China), which differs from previous UASs, and consists of an expansion tube, expansion tube connector, sheath tube, and operating handle (Figure 1). The longitudinal slit on the operating handle is a pressure-regulating vent. A stone collection bottle is connected to the UAS and the central negative pressure suction of the operating room. The main advantages of this V-UAS are its effective reduction of the intrarenal pressure and improved SFR. Flexible ureteroscopy (FURS) combined with V-UAS could become a new therapeutic partner for RIRS. Studies using V-UAS to treat infectious upper ureteral stones are lacking. Therefore, this prospective, randomized trial compared the efficacy and safety of RIRS with V-UAS and MPCNL in treating 1–2 cm infectious upper ureteral stones.

## 2. Methods

### 2.1. Patients

**Figure 2:**
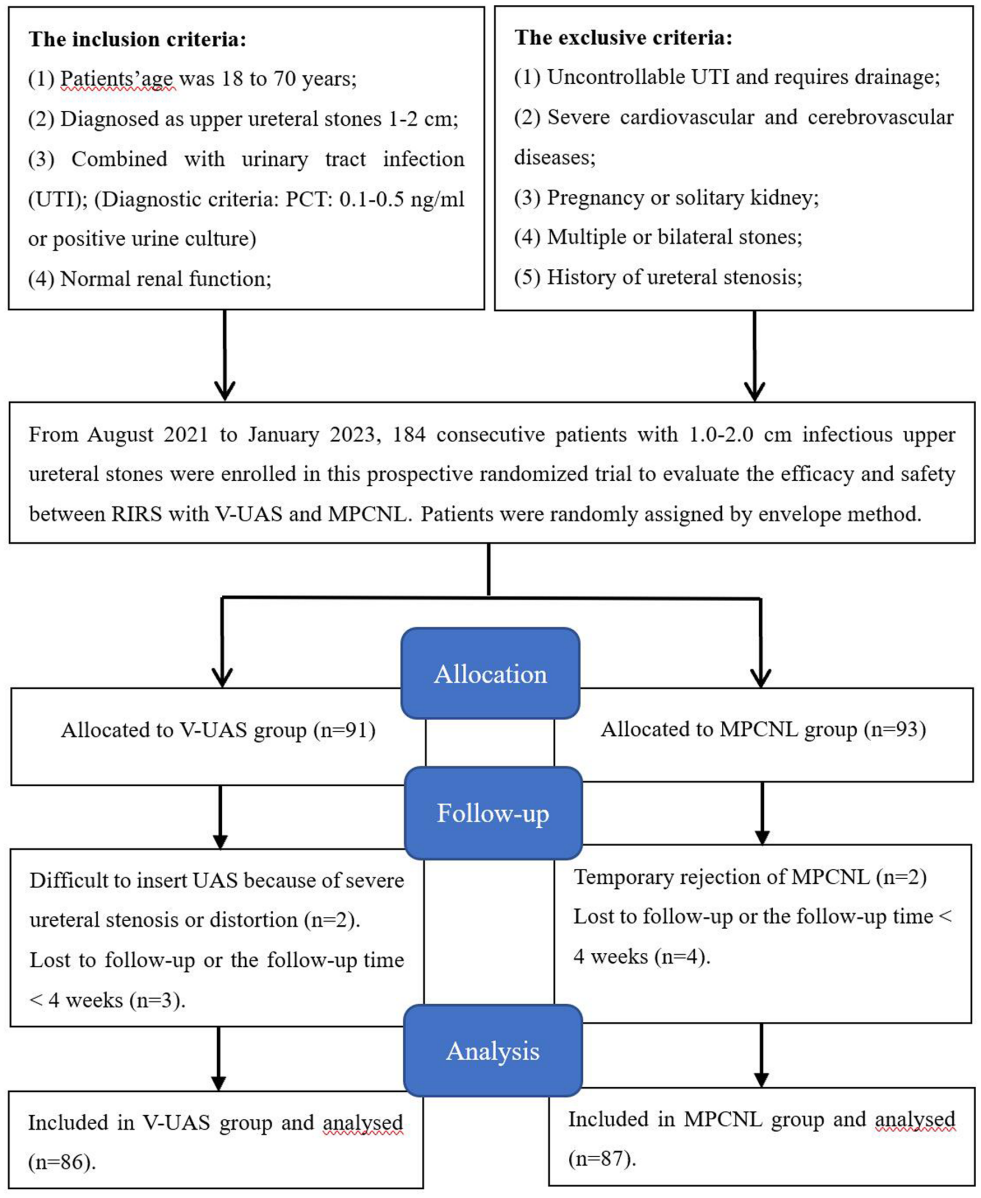
Flowchart for cases selection.

Patients with 1–2 cm infectious upper ureteral stones referred to our institute between August 2021 and January 2023 were considered for this study. Applying strict inclusion criteria, the patients were randomly assigned to the treatment groups using the envelope method. The study included 173 patients, 86 in the V-UAS group and 87 in the MPCNL group, based on power analysis performed to estimate the sample size (Figure 2). The participants' pretreatment evaluation included medical history, physical examination, laboratory investigations [urine analysis, urine culture and/or sensitivity, complete blood count, blood urea nitrogen, and serum levels of creatinine, C-reactive protein (CRP), and procalcitonin (PCT)], and radiologic investigations. Patients with a known UTI received antibiotic treatment until infection control was achieved. The study was approved by the clinical research ethics committee of the Affiliated Jiangning Hospital of Nanjing Medical University (ethics approval number: 202100387). Written informed consent was obtained from all participants.

### 2.2. Perioperative and surgical procedures

All patients underwent a plain preoperative abdominal radiography of the kidneys, ureters, and bladder and unenhanced computed tomography to assess hydronephrosis and the size, location, and number of the stones. We usually performed preprocedural urine cultures and applied appropriate antibiotic therapy based on the culture-antibiogram test results. Patients with negative urine cultures were treated with broad-spectrum antibiotics before surgery. The opportunity for operation depended on a downward trend in infection indicators and a negative urine culture. Stone size was determined by measuring the longest axis on the preoperative radiographs (14). The study was double-blind, and all procedures were performed by the same urologist. The selection of the surgical method for the enrolled patients was random, excluding any artificial subjective factors.

#### 2.2.1. MPCNL group

The patient was placed in a lithotomy position under general anesthesia. A 6 Fr ureteral catheter (Boston Scientific, USA) was inserted through the target ureter up to the ureteropelvic junction. The patient was then turned to a prone position, and a percutaneous tract was punctured under ultrasonographic guidance with an 18-gauge coaxial needle (Cook Medical, USA) for lithotripsy. A posterior middle calyx puncture was preferred in most cases because this region is considered an avascular area of the kidney. The percutaneous tract was serially dilated using fascial dilators (Cook Medical, USA) up to 18 Fr when fluid efflux was seen. Subsequently, a semirigid ureteroscope (8–9.8 Fr; Richard Wolf GmbH, Knittlingen, Germany) was inserted and connected to the collecting system. The upper ureteral stones were fragmented using a 200 or 365 µm holmium laser fiber, set to 15–20 W based on the stone hardness. Small stone fragments were washed out through the sheath by retrograde irrigation. Fluoroscopic images were taken at the end of the procedure to assess stone clearance. A 6 Fr double-J stent (Bard, USA) was inserted into the ureter using a loach guidewire (Bard), and a 14 Fr nephrostomy tube was placed.

#### 2.2.2. V-UAS group

**Figure 3:**
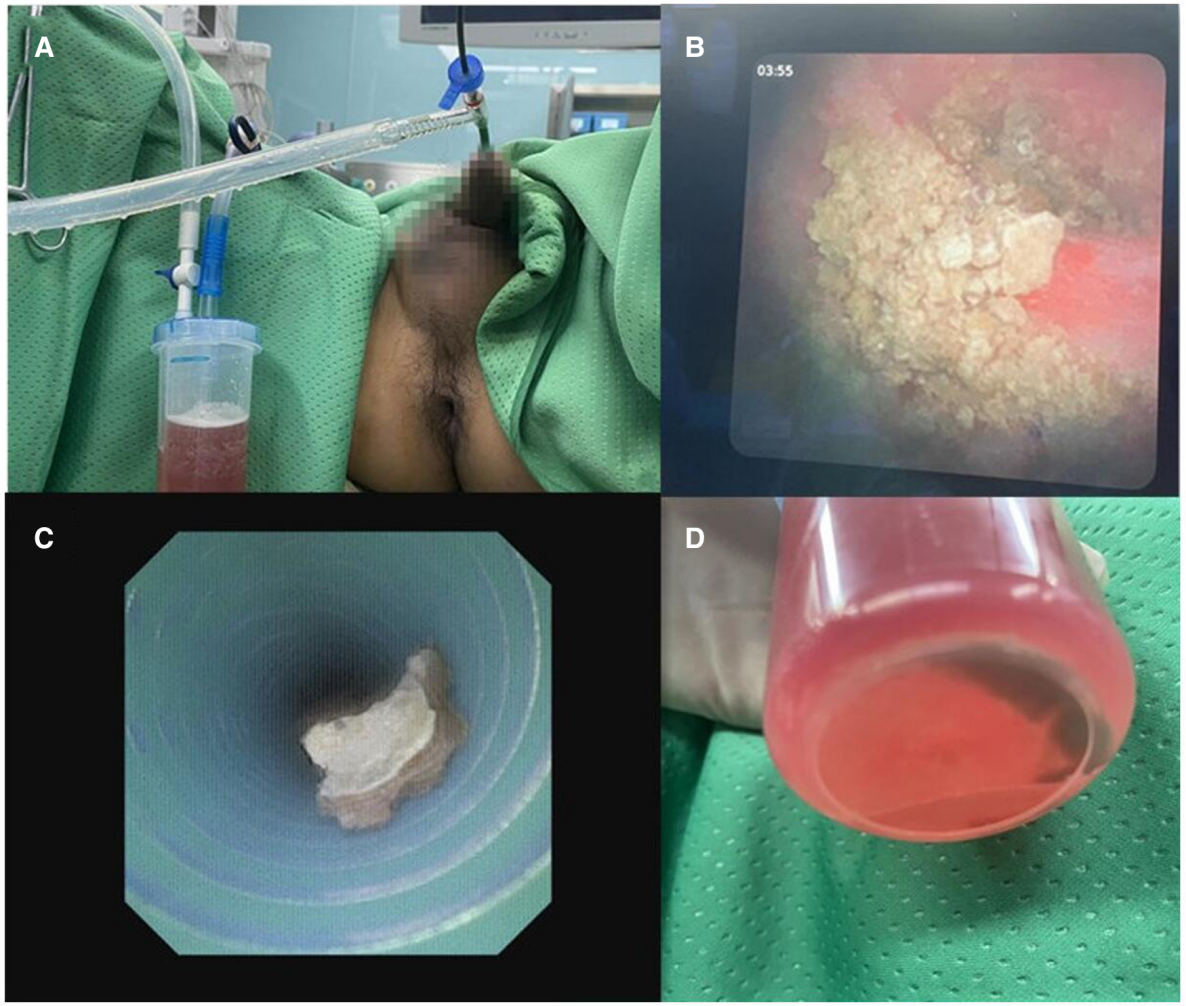
(**A**) Sucking out the stone fragments and infectious substances during lithortripsy by V-UAS; (**B**) stone fragments in renal pelvis; (**C**) fragments in the UAS by negative pressure suction; (**D**) stone collection bottle.

Under general anesthesia, the patient was placed in a lithotomy position for retrograde endoscopic access. A 0.032-inch loach guidewire was introduced into the upper urinary tract. This was followed by an 11/13 Fr V-UAS inserted into the upper diseased ureter. An 8 Fr electronic FURS (Woek Medical, Nanchang, China) was inserted through the V-UAS. We always pushed the stones into the renal pelvis or the middle or upper calyx after changing the position to head low and feet high to avoid dropped into the lower calyx. A complete inspection of the entire collecting system was performed and connected negative pressure to suck out infectious substances for urine culture if necessary. Then electronic FURS fragmented large stones by a 200 µm holmium laser fiber with the energy set to 0.8–1.0 J and the rate to 15–20 Hz. When possible, stone fragments were sucked out using the V-UAS (Figure 3). At the end of the procedure, the collecting system was visually reinspected for large stone fragments. The V-UAS and FURS were removed while visually assessing and documenting any ureteral injury. A 6 Fr double-J stent was placed in all patients at the end of the procedure. The patients were discharged three days after the operation when their condition was stable. If severe ureteral stenosis or distortion was encountered during the operation and it was difficult to insert the V-UAS, only a double-J stent was placed for dilation.

### 2.3. Postoperative follow-up

The patients were assessed for white blood cell (WBC) count, CRP and PCT at 6, 24, and 48 h postoperatively. If the stone composition was pure uric acid or cystine, so it could not be visualized in the kidneys, ureters, and bladder radiographs, a computed tomography examination was used to search for the residual stones. A stone-free status was defined as no radiological evidence of stones or the presence of ≤2 mm asymptomatic fragments in the urinary system (15–17). The primary study outcome was the SFR 1 day, two weeks, and four weeks postoperatively. The secondary endpoints were operative time, postoperative hospital stay, and operation-related complications. Patients with residual stones underwent auxiliary procedures four weeks or more after surgery. These included external physical vibration lithecbole (EPVL), extracorporeal shock wave lithotripsy (ESWL), or position therapy. The double-J stent was removed four weeks postoperatively.

### 2.4. Statistical analysis

IBM SPSS Statistics for Windows, Version 22.0 (IBM Corp., Armonk, NY, USA) was used for statistical analysis. Continuous variables are presented as means ± standard deviations. The groups were compared for patient demographics, follow-up, and surgical outcomes using independent samples *t*-test; The Shapiro-Wilk test was used to test the normality of the initial data. A chi-squared test compared the groups for other pre- and postoperative clinical characteristics. A *P*-value < 0.05 was considered statistically significant.

## 3. Results

### 3.1. Demographics and preoperative clinical characteristics

The 173 patients in this study were randomly assigned to the V-UAS (*n* = 86) or the MPCNL (*n* = 87) group. The groups were similar in the patients' demographics and preoperative clinical characteristics (Table 1). Similar mean stone size was found in the V-UAS (1.6 ± 0.4 cm) and MPCNL (1.5 ± 0.5 cm) groups (*P* = 0.148). The groups were also similar for preoperative infection indicators (WBC, CRP and PCT), mean age at diagnosis, body mass index, sex ratio, history of hypertension and diabetes, stone essence, degree of hydronephrosis, urine culture, and history of preoperative ESWL (all *P* > 0.05).

**Table 1:** Comparisons of patients’ demographics and preoperative clinical characteristics between two groups.

Variables, mean ± SD or *n* (%)	V-UAS group (*n* = 86)	MPCNL group (*n* = 87)	*P* value
Age, years	52.7 ± 9.3	51.3 ± 8.2	0.295
BMI, kg/m^2^	23.1 ± 3.9	22.6 ± 3.2	0.358
Gender			
Male	37 (43.0)	41 (47.1)	–
Female	49 (57.0)	46 (52.9)	0.588
Hypertension history			
No	44 (51.2)	39 (44.8)	–
Yes	42 (48.8)	48 (55.2)	0.404
Diabetes history			
No	53 (61.6)	58 (66.7)	–
Yes	33 (38.4)	29 (33.3)	0.489
Mean stone size, cm	1.6 ± 0.4	1.5 ± 0.5	0.148
Essence (Hounsfield units)	932.3 ± 107.9	856.4 ± 113.6	0.154
Hydronephrosis			
Mild	27 (31.4)	30 (34.5)	0.666
Moderate	36 (41.9)	38 (43.7)	0.809
Severe	23 (26.7)	19 (21.8)	0.452
Preoperative WBC, 10^9^/L	9.7 ± 4.1	9.3 ± 3.6	0.496
Preoperative CRP, mg/L	15.3 ± 5.4	16.1 ± 4.3	0.282
Preoperative PCT, ng/ml	0.103 ± 0.04	0.107 ± 0.03	0.457
Urine culture			
Negative	25 (29.1)	30 (34.5)	–
Positive	61 (70.9)	57 (65.5)	0.445
ESWL history			
No	29 (33.7)	33 (37.9)	–
Yes	57 (66.3)	54 (62.1)	0.564

BMI, body mass index; SD, standard deviation; WBC, white blood cell; CRP, C reactive protein; PCT, procalcitonin.

* *P *< 0.05.

** *P *< 0.01.

### 3.2. Postoperative clinical characteristics

Differences in postoperative clinical outcomes between the groups are shown in Table 2. The mean operative time in the V-UAS group was insignificantly longer than that in the MPCNL group (*P* > 0.05). The level of WBC, CRP and PCT in the V-UAS group at 24 and 48 h after surgery were significantly lower than in the MPCNL group (all *P *< 0.05). Moreover, there was no statistical significance between two groups in stone compositions (*P* > 0.05).

**Table 2:** Comparisons of surgical outcomes and postoperative clinical characteristics between two groups.

Variables, mean ± SD or *n* (%)	V-UAS group (*n* = 86)	MPCNL group (*n* = 87)	*P* value
Operative time, min	61.4 ± 5.2	60.3 ± 5.6	0.183
WBC, 10^9^/L			
Postoperative 6 h	14.4 ± 3.1	13.6 ± 3.8	0.131
Postoperative 24 h	15.5 ± 3.7	16.9 ± 3.0	0.007**
Postoperative 48 h	9.3 ± 2.2	10.0 ± 1.8	0.02*
Serum CRP concentration, mg/L			
Postoperative 6 h	62.5 ± 23.4	59.7 ± 12.3	0.325
Postoperative 24 h	67.6 ± 19.1	74.6 ± 10.2	0.003**
Postoperative 48 h	23.2 ± 11.6	26.7 ± 8.6	0.025*
Serum PCT concentration, ng/ml			
Postoperative 6 h	0.179 ± 0.042	0.174 ± 0.033	0.385
Postoperative 24 h	0.368 ± 0.093	0.421 ± 0.147	0.005**
Postoperative 48 h	0.095 ± 0.037	0.112 ± 0.058	0.022*
Initial SFS (postoperative 1 day)			
No	23 (26.8)	12 (13.8)	–
Yes	63 (73.2)	75 (86.2)	0.034*
SFS at the 2nd week end			
No	15 (17.4)	8 (9.2)	–
Yes	71 (82.6)	79 (90.8)	0.110
SFS at the 4nd week end			
No	5 (5.8)	4 (4.6)	–
Yes	81 (94.2)	83 (95.4)	0.719
Complications			
Fever	2 (2.4)	9 (10.3)	0.031*
Bleeding	1 (1.2)	3 (3.4)	0.317
Pain	2 (2.4)	13 (14.9)	0.003**
Urosepsis	0 (0.0)	4 (4.6)	0.044*
Stone compositions			
Calcium oxalate	17 (19.8)	19 (21.8)	0.737
Calcium phosphate	12 (14.0)	15 (17.3)	0.551
Struvite or carbonated apatite	53 (61.6)	51 (58.6)	0.686
Uric acid or cysteine	4 (4.6)	2 (2.3)	0.398
Postoperative hospital stay, days	3.7 ± 0.8	5.9 ± 0.5	<0.001**

SD, standard deviation; WBC, white blood cell; CRP, C-reactive protein; PCT, procalcitonin; SFS, stone-free status.

* *P *< 0.05.

** *P *< 0.01.

Postoperative 1 day the SFR in the V-UAS group was significantly lower than that in the MPCNL group (73.2% vs. 86.2%, *P* = 0.034). However, there was no statistical significance between two groups in SFRs during postoperative 2 weeks and 4 weeks (all *P* > 0.05). Postoperative complications, classified using the modified Clavien system (18, 19), included fever (≥38.5°C), bleeding, pain and urosepsis. In terms of the rates of fever, pain and urosepsis, MPCNL group were all significantly higher than those in the V-UAS group (10.3 vs. 2.4%, *P* = 0.031; 14.9 vs. 2.4%, *P* = 0.003; 4.6 vs. 0.0%, *P* = 0.044; respectively). No significant difference was found between two groups in bleeding. Meanwhile, postoperative hospital stay in the V-UAS group was more shorten than that in the MPCNL group (3.7 vs. 5.9 days, *P* < 0.001).

## 4. Discussion

Ureteral stones are a common urological disease. As people's living standards improve, the prevalence of ureteral stones in the population is increasing. Surgical treatment for ureteral stones aims to completely remove them, relieve the obstruction, and control infection with as few complications as possible (6). With the recent progress in technology and equipment, the surgical treatment for ureteral stones has developed from single open surgery to multiple minimally-invasive surgical approaches, primarily including ESWL, RIRS, and PCNL (20). The success rate of ESWL for ureteral stones is low due to the anatomical location of the ureter, which affects the localization of ureteral stones and the full utilization of shock wave energy (21). According to the European Association of Urology, RIRS and PCNL are recommended as alternatives to remove 1–2 cm infectious upper ureteral stones (6).

In the past decade, we have used MPCNL with an 18-Fr access tract to decrease the risk of complications encountered with standard PCNL. However, the technique is not faultless. Compared with RIRS, MPCNL has a higher risk of complications. Moreover, the single-tract MPCNL has more blind areas in the renal collecting system than RIRS. Therefore, we developed a new technique that combines RIRS with V-UAS to avoid the shortcomings of MPCNL and improve therapeutic outcomes of 1–2 cm infectious upper ureteral stone removal. This was the first study to compare the clinical outcomes between RIRS with V-UAS and MPCNL.

RIRS with V-UAS can help accelerate stone fragments' extraction. Wu et al. (22) compared in 2022 a novel vacuum suction ureteroscopic laser lithotripsy (URS) with the traditional URS for managing impacted upper ureteral stones. They concluded that vacuum suction URS was an effective modality for impacted upper ureteral stones, and has a shorter operating time, lower fever rate and a higher primary SFR compared with traditional URS. Our outcomes showed that the SFR in the V-UAS group at postoperative 1 day was significantly lower than that in the MPCNL group. However, there was no statistical significance between two groups in SFRs during postoperative 2 weeks and 4 weeks. Overall SFRs in two groups were similar after the follow-up period.

The complication rate in PCNL increases with the nephrostomy tract size (23, 24). MPCNL with an 18-Fr access tract decreases the percutaneous tract size and minimizes renal injury. Compared with 22–24 Fr access tracts used in standard PCNL, the 18 Fr access tract represents a 55.6% reduction in the nephrostomy tract surface area (25, 26). Therefore, our data showed no difference between the two groups in the bleeding rate. The reported infection complication rates for traditional RIRS and MPCNL range between 1.7 and 32.1% (27, 28). However, the incidence of this complication might be higher for infectious than non-infectious stones. To solve this problem and improve surgical safety, we adopted the novel V-UAS with RIRS. The second advantage of V-UAS was its potential to effectively prevent excessive intrarenal pressure by sucking out infectious substances during lithotripsy. Due to its simultaneous suction and continuous irrigation, it guarantees clear vision. It is worth mentioning that PCT is a highly sensitive and specific infection indicator, recognized as the most sensitive diagnostic indicator for urosepsis (29). Our study found that all infection indicators in the V-UAS group were lower than in the MPCNL group at the postoperative 24 h and 48 h. Therefore, the fever, pain, and urosepsis rates in the MPCNL group were significantly higher than those in the V-UAS group. Furthermore, the postoperative hospital stay in the MPCNL group was longer than that in the V-UAS group.

This study also had some limitations. The follow-up was short and may have affected the outcome. Furthermore, we did not use computed tomography in all patients during follow-up, which might have biased the diagnosis of residual stones. Finally, this was a single-center study with a small sample. There may have been a certain amount of sampling error. Therefore, large-scale multicenter prospective studies are required to prove our conclusions. The ideal procedure will likely be formulated through a long period of clinical applications and observations.

## 5. Conclusions

Our study showed that RIRS with V-UAS, a new partnership to treat 1–2 cm infectious upper ureteral stones, was satisfying as it achieved a high SFR rate and a low rate of infectious complications. This method was safe and reproducible in clinical practice. However, therapeutic plans need to be individualized and perfected according to other factors such as economic burden, prevailing custom, patient's wishes, and stone essence.

## Data Availability

The raw data supporting the conclusions of this article will be made available by the authors, without undue reservation.
